# Ab *initio* molecular dynamics simulation of low energy radiation responses of α-Al_2_O_3_

**DOI:** 10.1038/s41598-017-03827-1

**Published:** 2017-06-15

**Authors:** Y. G. Yuan, M. Jiang, F. A. Zhao, H. Chen, H. Gao, H. Y. Xiao, X. Xiang, X. T. Zu

**Affiliations:** 1 Institute of Nuclear Physics and Chemistry, China Academy of Engineering Physics, Mianyang, 621900 China; 2 School of Physical Electronics, University of Electronic Science and Technology of China, Chengdu, 610054 China; 3 Institute of Fundamental and Frontier Sciences, University of Electronic Science and Technology of China, Chengdu, 610054 China

## Abstract

In this study, an *ab initio* molecular dynamics method is employed to investigate the response behavior of α-Al_2_O_3_ to low energy irradiation. Different from the previous experiments, our calculations reveal that the displacements of oxygen dominate under electron irradiation and the created defects are mainly oxygen vacancy and interstitial. The experimental observation of the absorption peaks appearing at 203, 233 and 256 nm for α-Al_2_O_3_ under electron irradiations should be contributed by the oxygen defects and these defects will reduce the transmittance of α-Al_2_O_3_, which agrees well with the very recent experiment. This study demonstrates the necessity to reinvestigate the threshold displacement energies of α-Al_2_O_3_, and to introduce recombination center for oxygen defects to improve its optical properties and performance under radiation environment.

## Introduction

The sapphire phase of alumina (α-Al_2_O_3_) is widely used for numerous industrial applications, such as catalyst support, solid state laser and photovoltaic devices^1, 2^. Due to its high radiation resistance and wide band gap, α-Al_2_O_3_ is also used as an effective detector of ionizing radiation^2^. Its potential applications also include components of breeder blanket and diagnostic windows, as well as coating in future fusion reactors to avoid the permeation of light gases^3^. In these applications, α-Al_2_O_3_ is exposed to different kinds of radiation environment, i.e., neutron, low and swift heavy ions and γ-ray radiation. This leads to the generation, migration and aggregation of defects or defect clusters, which ultimately may deteriorate the mechanical properties of materials and influence their performance. It is of crucial importance to investigate the phase stability of α-Al_2_O_3_ under radiation and the underlying mechanism for defect generation to enhance its radiation tolerance and improve its performance.

In the past decades, the radiation damage effects of α-Al_2_O_3_ have been extensively studied both experimentally and theoretically^4–16^. The sapphire crystal of Al_2_O_3_ was irradiated by the ^238^U ions at temperatures around 80 K by Canut *et al*.^5^. The lattice disorder induced by collective electronic excitation was confirmed by the Rutherford backscattering spectrometry in channeling geometry (RBS-C) analysis, and the optical absorption spectroscopy of α-Al_2_O_3_ exhibited the characteristic bands associated with oxygen vacancies^5^. Single crystalline α-Al_2_O_3_ samples were implanted at room temperature with 160 keV Pt^+^ ions by Alves *et al*., who found that 80% of the implanted ions occupied the aluminum lattice site, and the increase in doses made the implanted region highly damaged^6^. Kabir *et al*. irradiated the α-Al_2_O_3_ with 0.7 MeV ^129^Xe ions with fluences ranging from 5 × 10^11^ to 2 × 10^14^ ion/cm^2^, and characterized the samples with RBS-C measurements^4^. The RBS-C analysis suggested the presence of two incident ion effects on α-Al_2_O_3_ samples^4^: the creation of partial disorder that saturated at ~ 40% with a damage cross section of 7 × 10^−14^ cm^−2^, followed by a complete disorder starting from the surface and appearing at fluence larger than ~1.2 × 10^13^ ion/cm^2^. Microstructural evolution in crystalline α-Al_2_O_3_ during Xe^+^ ion irradiation has been investigated by Okubo *et al*., who found that the swift heavy ion irradiation caused lattice expansion and the structural modification led to structural amorphization above the energy around 100 MeV^10^. Kulkarni *et al*. irradiated the sapphire with various thermal neutron fluences in the range of 10^14^ to 10^18^ n/cm^2^, and found that the neutron irradiation introduced considerable oxygen vacancies, as confirmed by the presence of F (203 nm) and F^+^ (225 and 255 nm) bands in the optical absorption spectra^7^. Izerrouken *et al*. investigated the formation of color centers in α-Al_2_O_3_, and found that the content of single point defect (F^+^ center) and defect cluster (F_2_, F_2_^+^, F_2_^2+^) increased linearly with the increasing fast neutron fluences^8^. Similar results were also reported in the case of 90 MeV Xe^+^ ion irradiation^9^, where F^+^ center concentration monotonously increased with the ion fluence in the range up to 10^13^ Xe/cm^2^.

Theoretically, Williford *et al*. have investigated the displacement energies of α-Al_2_O_3_ using classical molecular dynamics (MD) method^13^. On the other hand, several density functional theory (DFT) calculations have been carried out to investigate the defect formation and migration in α-Al_2_O_3_^3, 14, 16^. In spite of these studies, there still lacks of an atomic-level understanding of the mechanisms for defect generation in α-Al_2_O_3_, as well as the defect distribution and the interaction between defects. In recent years, the ab *initio* molecular dynamics (AIMD) method has been widely employed to simulate the low energy recoil events in ceramic materials like pyrochlores, fluorite-structure oxides and carbides, in which a number of new defective states and new mechanisms for defect generation that are different from classical MD have been predicted^17–20^. In this study, the AIMD method is employed to investigate the radiation responses of α-Al_2_O_3_ to low energy irradiation. The threshold displacement energies, the pathway for defect generation, the type of created defects, the role of charge transfer during the dynamic process, as well as the impact of created defects on the electronic structure of α-Al_2_O_3_, all have been provided. The presented results will be useful for understanding the structure-property relationship of α-Al_2_O_3_ and improving its properties and performances for its application as the substrate material for GaN growth for the production of blue light-emitting diode (LED), thin film passivation material for high-efficiency solar cells, luminescence dosimetry, and so on.

## Results and Discussion

### Ground-state properties of α-Al_2_O_3_

The structural and elastic properties of α-Al_2_O_3_ are first calculated and compared with experimental and other theoretical data. The optimized lattice constants for α-Al_2_O_3_ are listed in Table 1, which agree well with the theoretical^21^ and experimental^22^ results. The bulk (B), shear (G) and Young’s (E) modulus for α-Al_2_O_3_ are determined to be 264.3, 166.3 and 412.4 GPa, respectively. It appears that our results are in good agreement with the experimental results^23^ and are comparable with the theoretical values^24^ reported by Holm *et al*. The calculated Poisson’s ratio for α-Al_2_O_3_ of 0.24 also agrees well with the experimental^23^ and other theoretical results^24^.

**Table 1:** Calculated lattice constant (Å), elastic moduli (GPa) and Poisson’s ratio (σ) for α-Al_2_O_3_.﻿ ﻿B: bulk modulus, G: shear modulus. E: Young's modulus.

	Lattice Constant	B	G	E	σ
Our cal.	(4.81,4.81,13.14)	264.3	166.3	412.4	0.24
Other cal.	(4.80,4.80,13.11)^a^	246.4^c^	158.6^c^	390.0^c^	0.24^c^
Exp.	(4.77,4.77,13.01)^b^	255.0^d^	165.3^d^	404.6^d^	0.23^d^

^a^Ref. 21.

^b^Ref. 22.

^c^Ref. 24.

^d^Ref. 23.

### The threshold displacement energies in α-Al_2_O_3_

The threshold displacement energy (E_d_), which is defined as the minimum transferred kinetic energy for primary knock-on atom (PKA) to be permanently displaced from its lattice site, is one of the critical physical parameters for estimating damage production rates and predicting the defect profile under electron, neutron and ion irradiation^19, 25^ The calculated E_d_s for O and Al recoils along different directions in α-Al_2_O_3_ are summarized in Table 2.

**Table 2:** Threshold displacement energies (E_d_) for O and Al recoils. The minimum values for O and Al PKAs are indicated in bold.

Direction	E_d_ (eV)	
	O recoils	Al recoils
[0001]	32.5	**47.5**
$[\overline{1}2\overline{1}0]$	40.5	148
$[2\overline{1}\overline{1}0]$	27	105, 51.4^a^
$[11\overline{2}0]$	**25**	74.5
$[11\overline{2}3]$	35	107.5
$[10\overline{1}0]$	30, 54.3^a^	58, 27.7^a^
$[11\overline{2}6]$	27	114
$[01\overline{1}2]$	51.5	>150
$[\overline{2}4\overline{2}3]$	76	>150
$[2\overline{1}\overline{1}3]$	29	113
$[01\overline{1}1]$	34.5	87.5
$[22\overline{4}1]$	30	85
$[02\overline{2}1]$	27	66.5
$[11\overline{2}2]$	30.5	105.5
$[41\overline{5}6]$	31.5	>150
$[44\overline{8}3]$	39	71

^a^Ref. 13.

For the oxygen recoils, the maximum and minimum E_d_ values are determined to be 76 and 25 eV for $[\overline{2}4\overline{2}3]$ and $[11\overline{2}0]$ directions, respectively, as shown in Table 2. The defect configurations for O $[\overline{2}4\overline{2}3]$ and O $[11\overline{2}0]$ are both oxygen vacancy and oxygen dumbbell pair, whereas the defect generation pathways show different character. In the case of $[\overline{2}4\overline{2}3]$, the oxygen atom moves 3.63 Å away from its lattice site and forms a dumbbell pair with its neighboring oxygen atoms. As for O $[11\overline{2}0]$, the oxygen atom moves 2.20 Å to collide with its neighboring oxygen atom and occupies its lattice site. The struck oxygen atom moves 1.9 Å away from its equilibrium site and forms a dumbbell pair with the neighboring oxygen atoms eventually. On the other hand, the minimum E_d_ value for Al PKA is 47.5 eV along the [0001] direction, i.e., the Al atom along this direction is the most likely to be displaced. In the cases of $[01\overline{1}2]$, $[\overline{2}4\overline{2}3]$ and $[41\overline{5}6]$ for Al PKA, the E_d_s are predicted to be larger than 150 eV, i.e., the PKA is not permanently displaced at energy up to 150 eV. Among the determined threshold displacement energies, the E_d_ value of 148 eV for Al $[\overline{1}2\overline{1}0]$ is the largest. The associated defects and the pathway for defect generation for Al [0001] and Al $[\overline{1}2\overline{1}0]$ recoil events are found to be somewhat different. In the case of Al [0001], the Al PKA moves 1.86 Å away from its lattice site to eject its neighboring aluminum atom and occupies its lattice site. Then, the collided aluminum atom moves along the [0001] direction to hit another neighboring Al atom, and forms an interstitial occupying the octahedral site. The third collided aluminum atom also forms an interstitial occupying the octahedral site, which is 2.01 Å away from its original site. As a result, the final defect structure consists of two aluminum Frenkel pairs (FPs). As for Al recoil along the direction of $[\overline{1}2\overline{1}0]$, besides the Al PKA, its neighboring oxygen atom is also involved in the displacement events. Consequently, the damage end state contains one aluminum interstitial occupying the octahedral site, one aluminum vacancy, one oxygen dumbbell and one oxygen vacancy. These results suggest that the displacement events in α-Al_2_O_3_ are strongly dependent on the recoil direction and the species of the PKA.

**Figure 1:**
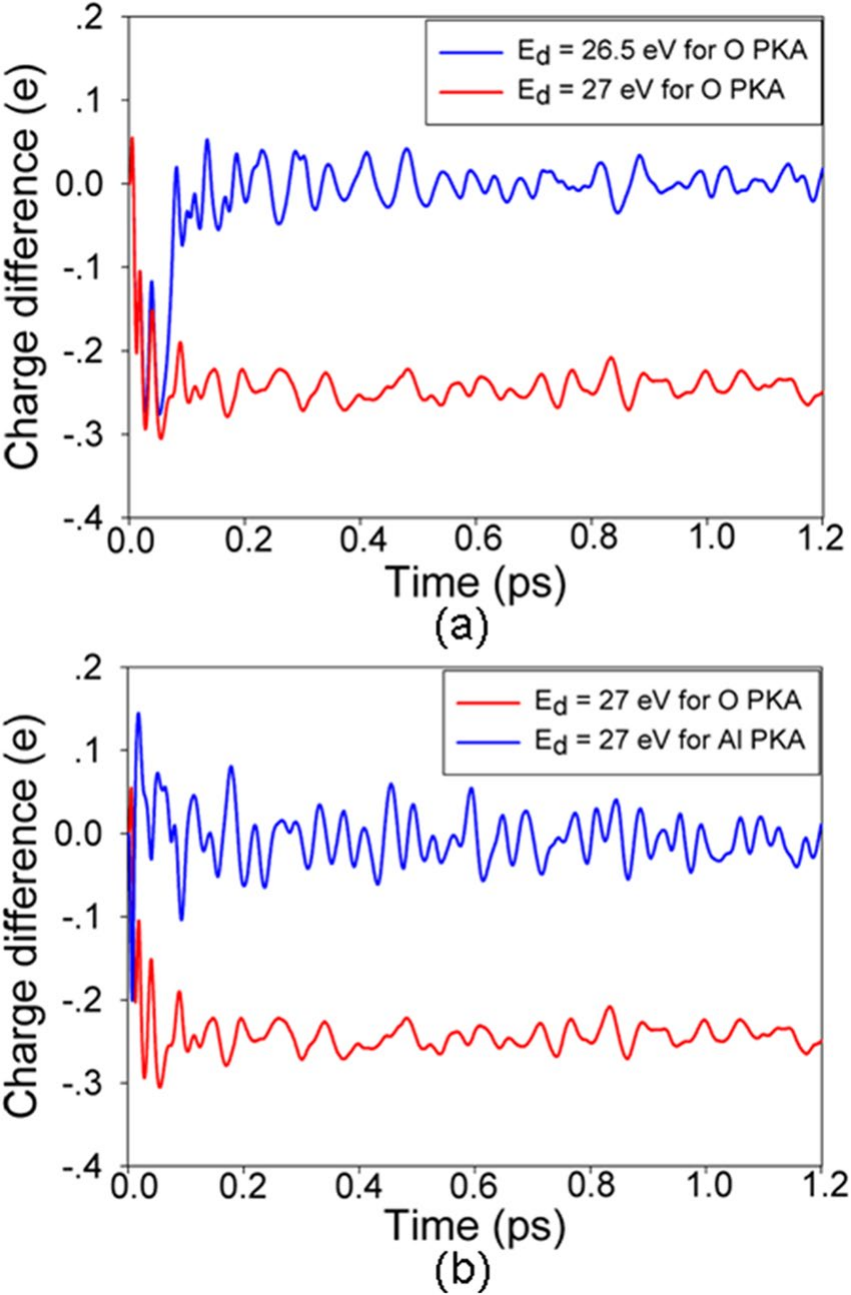
Charge difference for (**a**) O $[2\overline{1}\overline{1}0]$ PKA at energies of 26.5 and 27 eV; (**b**) O and Al PKAs along the $[11\overline{2}0]$ direction at the energy of 27 eV.

**Figure 2:**
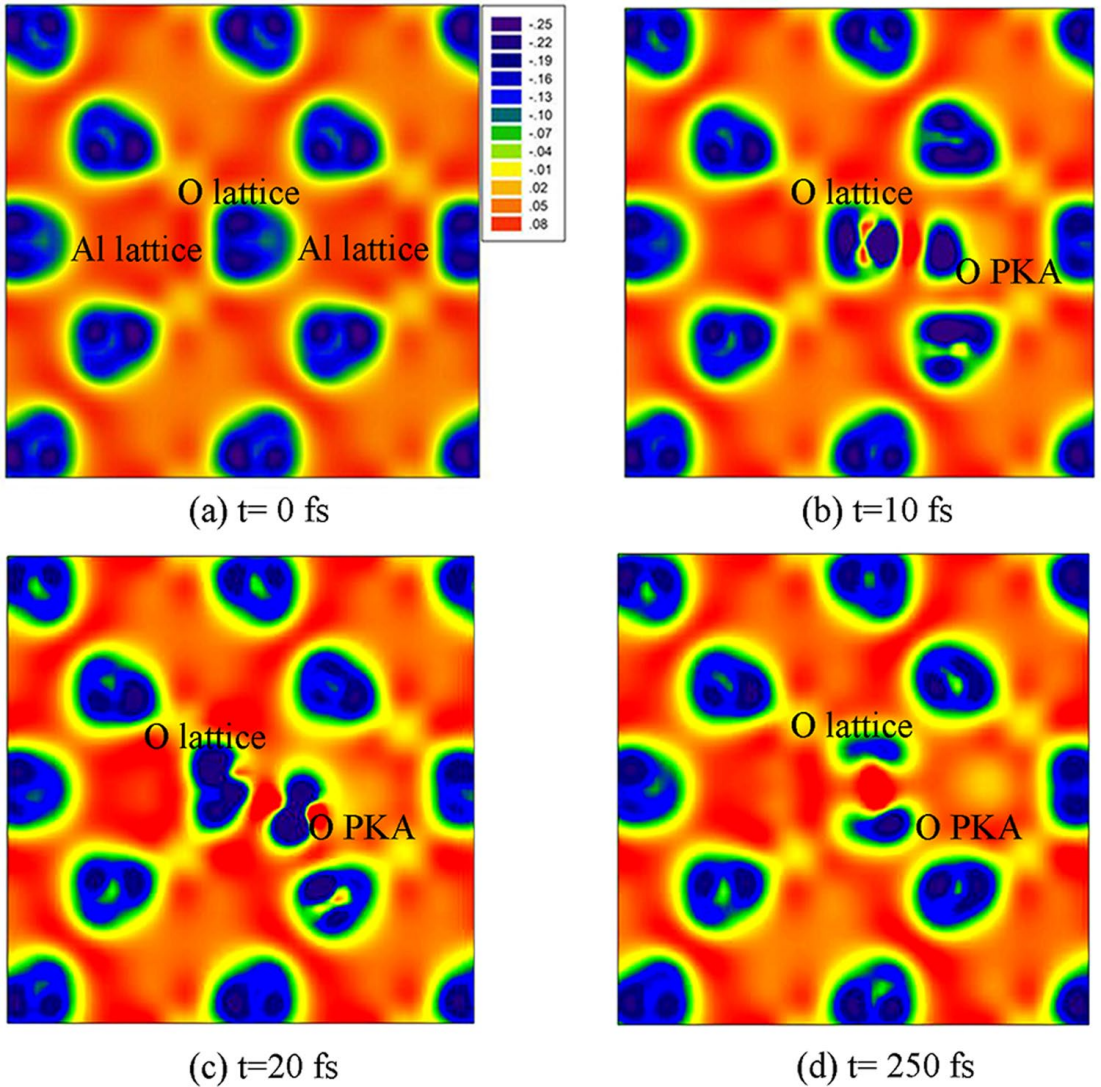
Charge-density contours projected onto plane during O $[2\overline{1}\overline{1}0]$ recoil events at energy of 27 eV.

Theoretically, Williford *et al*. have studied the displacement energies of α-Al_2_O_3_ employing the classical molecular dynamics (MD) method^13^. It is found that the E_d_s of 51.4 eV for Al $[2\overline{1}\overline{1}0]$ and 27.7 eV for Al $[10\overline{1}0]$ obtained by the MD method are much smaller than our AIMD results. In the previous AIMD simulation of low energy recoil events in SiC and pyrochlores^18–20, 25^, it is also found that the threshold displacement energies and the mechanism for defect generation obtained by AIMD method are generally different from the classical MD results. This may be due to the fact that recoil events are dynamic charge transfer processes, while such charge transfer was not considered in classical MD^26^. In this study, the charge transfer for the O and Al PKAs along the $[2\overline{1}\overline{1}0]$ direction during the displacement process as a function of time is illustrated in Fig. 1. In Fig. 1a, the variations of charge difference for O $[2\overline{1}\overline{1}0]$ at the energies of 27 and 26.5 eV are compared, which shows that charge transfer from and to the recoil atom takes place during the whole dynamics process. Especially, the charge changes at 27 eV are more significant than those at 26.5 eV, corresponding to reaching a stable defective state and returning to its lattice site, respectively. The Fig. 1b shows the variation in charge difference for Al and O recoils along the $[2\overline{1}\overline{1}0]$ direction at 27 eV. For Al PKA, the charge changes slightly and no defects are formed, whereas a relatively more significant charge transfer occurs for the O PKA. The charge-density contours projected onto $(2\overline{1}\overline{1}0)$ plane during O $[2\overline{1}\overline{1}0]$ recoil events at energy of 27 eV are illustrated in Fig. 2. Taking the initial charge-density (as shown in Fig. 2a) as a reference, it is clear that electron cloud deformation and charge redistribution takes place in the whole process. In Fig. 2b, when the O PKA is displaced to the interstitial site, it interacts with its neighboring atoms and the electron clouds around the lattice O start to deform toward the O PKA. With time evolution, there is a more significant electron cloud deformation to overcome the energy barrier for stable defect formation. The charge redistribution eventually leads to the formation of one O-O dumbbell, as shown in Fig. 2d.

Under electron irradiation the maximum energy transferred to an atom can be expressed as $T=2{E}_{e}({E}_{e}+2{m}_{e}{c}^{2})/M{c}^{2}$, where *E*_*e*_ is the incident energy, *m*_*e*_ is the electronic mass, *M* is the atomic mass and *c* is the velocity of light^25^. Thus, our calculated minimum E_d_ values of 25 eV for O recoil and 47.5 eV for Al recoil correspond to 152 and 415 keV electron irradiation, respectively. These radiation energies are comparable with the experimental measurements^27, 28^, while discrepancies exist in the threshold displacement energies. Arnold and Compton suggested that at 77 K the threshold radiation energy for α-Al_2_O_3_ was 430 keV, and the E_d_ values were 90 ± 5 for O ions and 50 ± 5 eV for Al ions^27^. In their study, the crystal orientation was not considered and the results were strongly dependent on the temperature, i.e., irradiation at 77 K produced many more centers than did a comparable irradiation at 300 K and the ratio of the yields at these two temperatures was at least ten^27^. Das reported that the threshold voltage was orientation dependent, which was 300 keV for [0001] and 240 keV for $[11\overline{2}0]$ direction^29^. Consequently, the predicted E_d_ values were 32 and 24 eV for Al [0001] and Al $[11\overline{2}0]$, respectively, and 53 and 41 eV for O [0001] and O $[11\overline{2}0]$, respectively^29^. In our study, the threshold displacement energies are determined by the creation of point defects such as oxygen or aluminum Frenkel pair. These defects may be too few to be observed experimentally, for which the threshold displacement energies were determined by the first appearance of defect clusters. Pells and Stathopoulos^30^ carried out high voltage electron microscope measurement with optical measurement of electron-irradiation induced color centers on α-Al_2_O_3_. They demonstrated that the threshold radiation energies were independent of the temperature and they were 400 ± 20 and 175 ± 20 keV for the oxygen and aluminum ions, respectively, and the determined E_d_ values were 76 ± 3 eV for O and 18 ± 3 eV for Al^30^. In their work, the crystal orientation was also not considered. Considering that impurities or defects may exist in the sapphire sample and the sapphire has a large band gap, the impurities or defects would make charge transfer process more likely and consequently affect the optical absorption. This may partly cause discrepancy between their work and our simulation.

### The defect distribution in α-Al_2_O_3_ after recoil events

**Figure 3:**
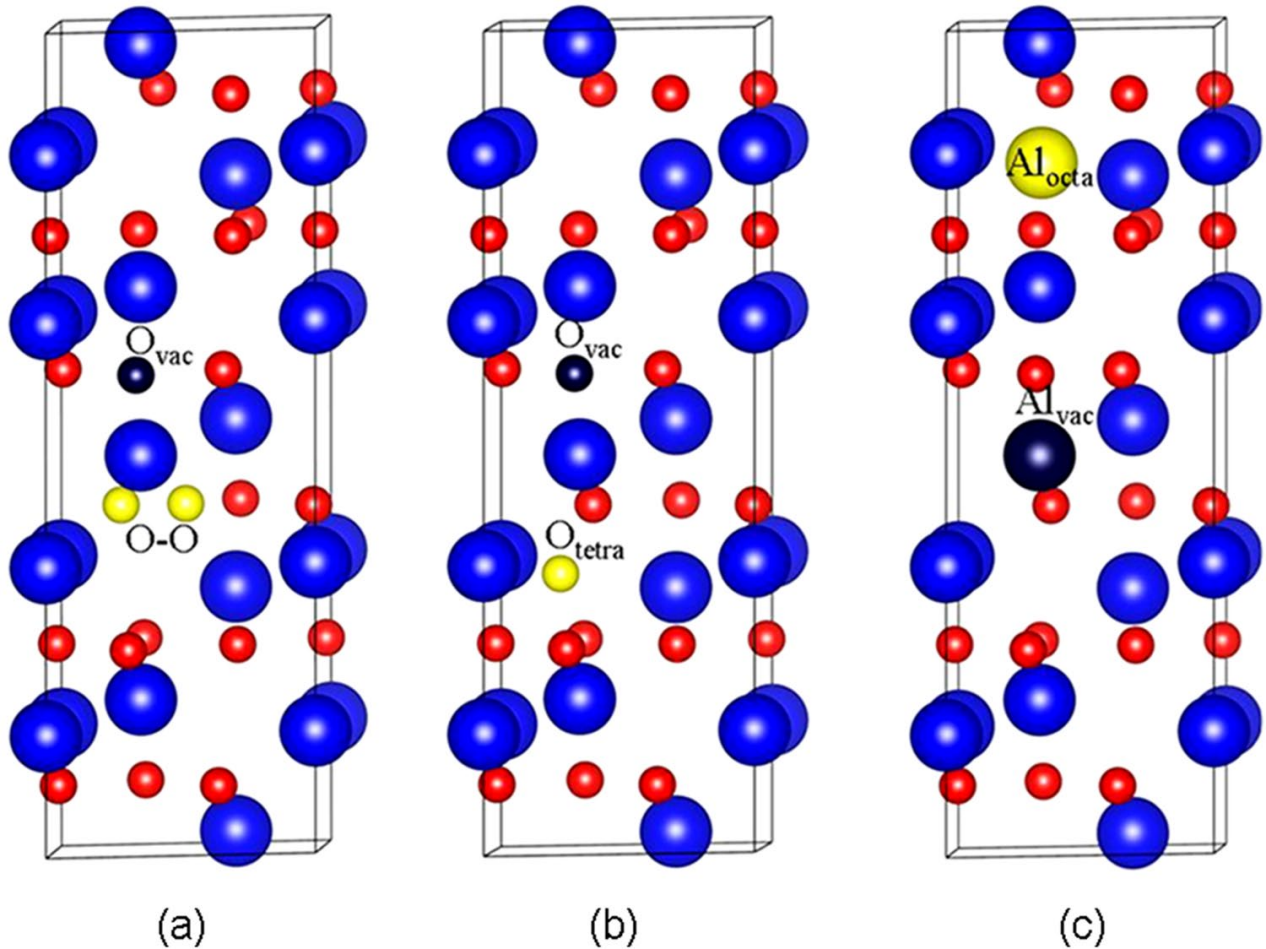
Illustration of schematic view of (**a**) O_vac_ + O-O dumbbell; (**b**) O_vac_ + O_tetra_ and (**c**) Al_vac_ + Al_octa_. The large blue and small red spheres represent the aluminum and oxygen atoms, respectively. The magenta large and small spheres represent the aluminum vacancy and oxygen vacancy, respectively. The yellow large and small spheres represent the aluminum interstitial and oxygen interstitial, respectively. O-O: oxygen dumbbell pair; O_tetra_: oxygen interstitial occupying the tetrahedral site; Al_octa_: aluminum interstitial occupying the octahedral site.

The defects created by the Al and O PKAs after recoil events are summarized in Table 3, and the defect configurations are illustrated in Fig. 3. As shown in the table, the damage end states after oxygen recoil events generally consist of one oxygen vacancy and one oxygen dumbbell pair with its neighboring oxygen atoms (see Fig. 3a), with the exception of O $[10\overline{1}0]$, O $[01\overline{1}2]$ and O $[02\overline{2}1]$. Despite the similar defect distribution, the pathways for defect generation behave different character and the displacement for O PKA is generally different from each other. In the cases of O $[10\overline{1}0]$, O $[01\overline{1}2]$ and O $[02\overline{2}1]$, the oxygen PKA moves away from their equilibrium sites, and finally occupies the tetrahedral site, as shown in Fig. 3b.

**Table 3:** The type of created defects and displacement (d_PKA_) for O and Al recoils.

	O		Al	
	Defect type	d_PKA_(Å)	Defect type	d_PKA_(Å)
[0001]	O-O + O_vac_	3.57	2Al_octa_ + 2Al_vac_	1.86
$[\overline{1}2\overline{1}0]$	O-O + O_vac_	4.51	Al_octa_ + Al_vac_ + O_vac_ + O_tetra_	4.89
$[2\overline{1}\overline{1}0]$	O-O + O_vac_	2.52	2Al_octa_ + 2Al_vac_	2.91
$[11\overline{2}0]$	O-O + O_vac_	2.20	Al_octa_ + Al_vac_	5.89
$[11\overline{2}3]$	O-O + O_vac_	3.85	2Al_octa_ + 2Al_vac_ + O_vac_ + O-O	3.63
$[10\overline{1}0]$	O_tetra_ + O_vac_	3.84	Al_octa_ + Al_vac_	2.39
$[11\overline{2}6]$	O-O + O_vac_	2.31	Al_octa_ + Al_vac_	6.43
$[01\overline{1}2]$	O_tetra_ + O_vac_	3.26	—	—
$[\overline{2}4\overline{2}3]$	O-O + O_vac_	3.63	—	—
$[2\overline{1}\overline{1}3]$	O-O + O_vac_	2.47	Al_octa_ + Al_vac_	6.57
$[01\overline{1}1]$	O-O + O_vac_	2.06	Al_octa_ + Al_vac_	5.43
$[22\overline{4}1]$	O-O + O_vac_	5.21	Al_octa_ + Al_vac_	5.58
$[02\overline{2}1]$	O_tetra_ + O_vac_	2.27	Al_octa_ + Al_vac_	5.99
$[11\overline{2}2]$	O-O + O_vac_	3.59	Al_octa_ + Al_vac_	5.15
$[41\overline{5}6]$	O-O + O_vac_	4.31		
$[44\overline{8}3]$	O-O + O_vac_	3.91	Al_octa_ + Al_vac_	5.26

O_vac_: oxygen vacancy; O_tetra_: oxygen interstitial occupying the tetrahedral site; O-O: oxygen-oxygen dumbbell pair; Al_vac_: aluminum vacancy; Al_octa_: aluminum interstitial occupying the octahedral site.

For aluminum recoil events, one aluminum vacancy (Al_vac_) and one aluminum interstitial occupying the octahedron site (Al_octa_) are created in most cases, as shown in Fig. 3c. Similar to the cases of oxygen recoil events, the mechanisms for defect creation and the displacement of Al PKA are generally different. In the cases of Al [0001] and Al $[2\overline{1}\overline{1}0]$, besides the Al PKA, another lattice Al is also displaced to the octahedral site and eventually two pairs of aluminum FP are formed. For Al $[\overline{1}2\overline{1}0]$, the formation of Al FP is accompanied by the involvement of lattice oxygen, which moves to the tetrahedral site and leads to the formation of one oxygen Frenkel pair. The most complex defect configuration is found to be the case of Al $[11\overline{2}3]$, for which two Al FPs, one oxygen vacancy and one O-O dumbbell are formed.

In the literature, the damage end states for α-Al_2_O_3_ as a result of exposure to radiation have been reported to be aluminum and oxygen vacancy^14, 31–34^, interstitial atom^14, 31, 33^, or complementary Frenkel pairs in two sublattice^14, 34^, agreeing well with our AIMD calculations. Platonenko and Zhukovskii *et al*. demonstrated that the oxygen interstitial could migrate above certain temperature and form the dumbbell pair (O_reg_−O_i_) in α-Al_2_O_3_^3, 14^, which is also in good agreement with our simulations.

### Impact of point defects on the electronic structure of α-Al_2_O_3_

**Figure 4:**
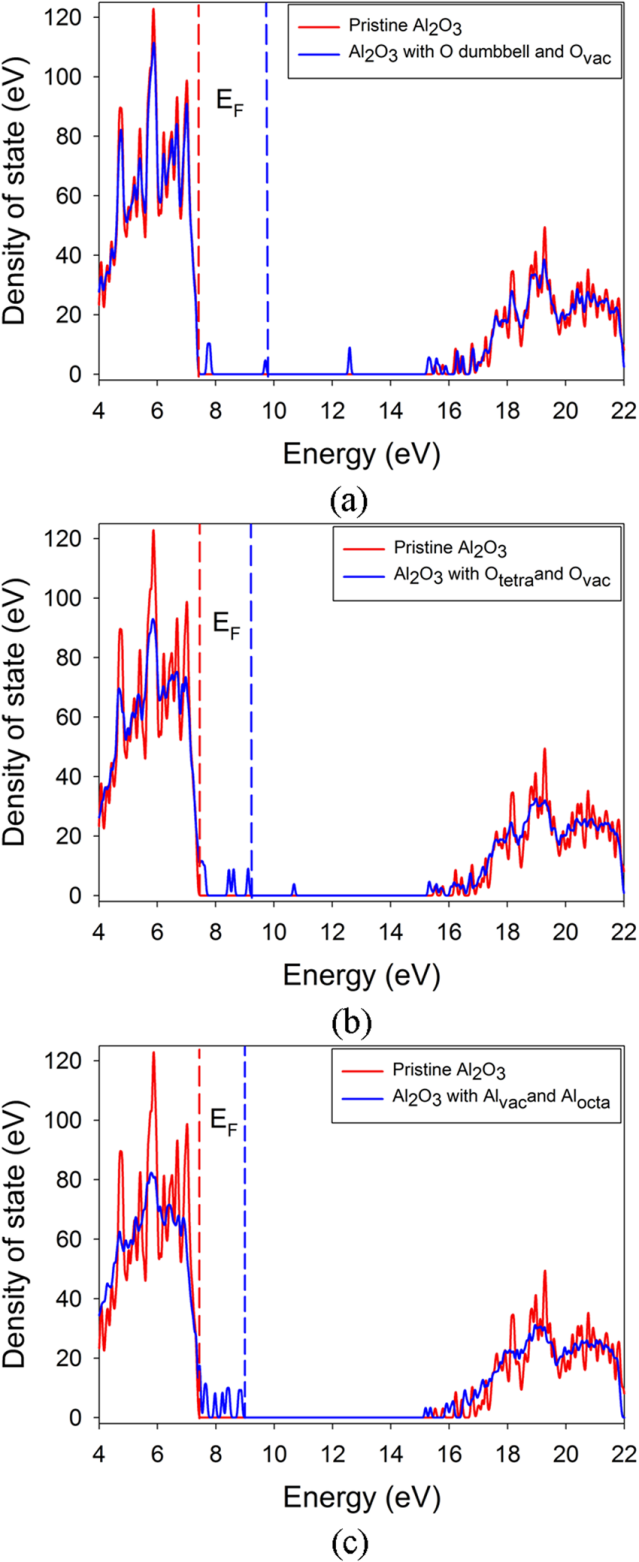
Total density of state distribution for (**a**) α-Al_2_O_3_ with oxygen vacancy and oxygen dumbbell pair; (**b**) α-Al_2_O_3_ with oxygen vacancy and oxygen interstitial occupying the tetrahedral site; (**c**) α-Al_2_O_3_ with aluminum vacancy and aluminum interstitial occupying the octahedral site. E_F_ indicates the Fermi level.

Aluminum oxide is one of the earlier materials used in luminescence dosimetry, while the application is limited by its low thermo-luminescence sensitivity. Great efforts have been devoted to study the thermo-luminescence and optical stimulated luminescence sensitivity, the electronic structure as well as the optical absorption of α-Al_2_O_3_. In order to explore how the radiation damage influences the band gap and optical absorption, first-principles calculations based on density functional theory are further carried out to study the electronic structures of damaged α-Al_2_O_3_. As discussed above, the associated defects are mainly aluminum FP and oxygen FP. The total density of state distribution of damaged α-Al_2_O_3_ with oxygen FP and aluminum FP are analyzed and compared with that of pristine state in Fig. 4. The band gap for pristine sapphire phase of alumina is predicted to be 7.7 eV, which is much smaller than the experimental data of 8.7 eV^35^. This underestimation is because of the well-known discontinuity of exchange-correlation energy of the local-density approximation. For the damaged α-Al_2_O_3_ with O_vac_ + O-O dumbbell and O_vac_ + O_tetra_, the Fermi level shifts from 7.6 eV to the higher energy value of 9.9 and 9.3 eV, respectively. Besides, defect levels are observed near the valence band maximum (VBM) and in the forbidden band region. Obviously, the presence of point defect influences the electronic structure of α-Al_2_O_3_ significantly, indicating that the optical absorption of α-Al_2_O_3_ under irradiation will be affected. As for α-Al_2_O_3_ with Al_vac_ + Al_octa_, the defect levels appear around the VBM and conduction band minimum.

**Figure 5:**
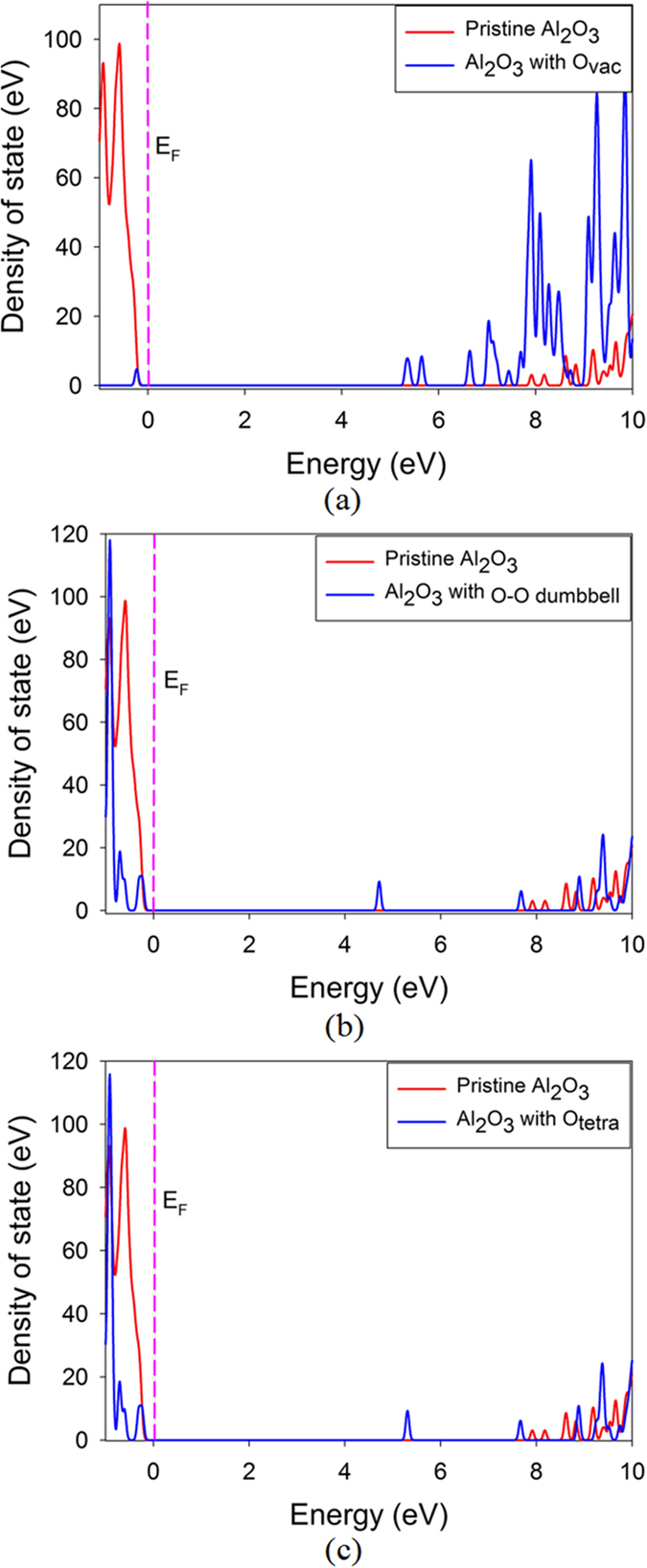
Total density of state distribution for α-Al_2_O_3_ with (**a**) oxygen vacancy; (**b**) oxygen dumbbell pair; (**c**) oxygen interstitial occupying the tetrahedral site. E_F_ indicates the Fermi level and is set to be zero.

To identify the contribution of the defect to optical absorption, we further analyze the electronic structures of defective α-Al_2_O_3_ with single O vacancy and interstitial, as illustrated in Fig. 5. Here, the Al oxygen and interstitial are not considered because the Al recoils have generally much larger threshold displacement energies than oxygen atoms and the oxygen defects dominate under electron irradiation. For α-Al_2_O_3_ with O_vac_, there are two defect levels appearing at 5.35 and 5.65 eV, corresponding to the wavelengths of 232 and 219 nm within the ultraviolet (UV) light region, respectively. In the cases of α-Al_2_O_3_ with O-O dumbbell and O_tetra_, the induced defect levels are located at 4.73 eV (262 nm) and 5.27 eV (235 nm), respectively. Wang *et al*. investigated the optical properties of α-Al_2_O_3_ under 1.2 MeV electron irradiations using the optical absorption spectra, and found that the absorption peaks mainly appeared at the wavelength of 203, 233 and 256 nm^36^. Our calculations show that these peaks should be contributed by the oxygen vacancy and interstitial defects. Obviously, the presence of these defects will reduce the transmittance of α-Al_2_O_3_. Ke *et al*. investigated the change of the surface roughness and transmittance caused by electron and proton irradiation, and found that the transmittance of α-Al_2_O_3_ decreased most remarkably in the ultraviolet band under the 100 ~ 150 keV electron irradiation^37^. This is also in good agreement with our simulations. These results suggest that it is necessary to enhance the radiation tolerance of α-Al_2_O_3_ or introduce recombination center for oxygen defects to improve its optical properties and performance under radiation environment.

## Conclusions

In summary, low energy recoil events in α-Al_2_O_3_ have been investigated by an *initio* molecular dynamics method. Generally, the threshold displacement energies for oxygen are smaller than those for aluminum, indicating that the displacement of oxygen dominates under electron irradiation and the created defects are mainly oxygen vacancy and interstitials. Moreover, the oxygen interstitial forms the dumbbell defect configuration or occupies the tetrahedral site. For α-Al_2_O_3_ with O_vac_, there are two defect levels appearing at 232 and 219 nm within the ultraviolet (UV) light region. In the cases of α-Al_2_O_3_ with O-O dumbbell and O_tetra_, the induced defect levels are located at 262 nm and 235 nm, respectively. These defects will influence the optical absorption and reduce the transmittance of α-Al_2_O_3_ significantly.

## Methods

**Figure 6:**
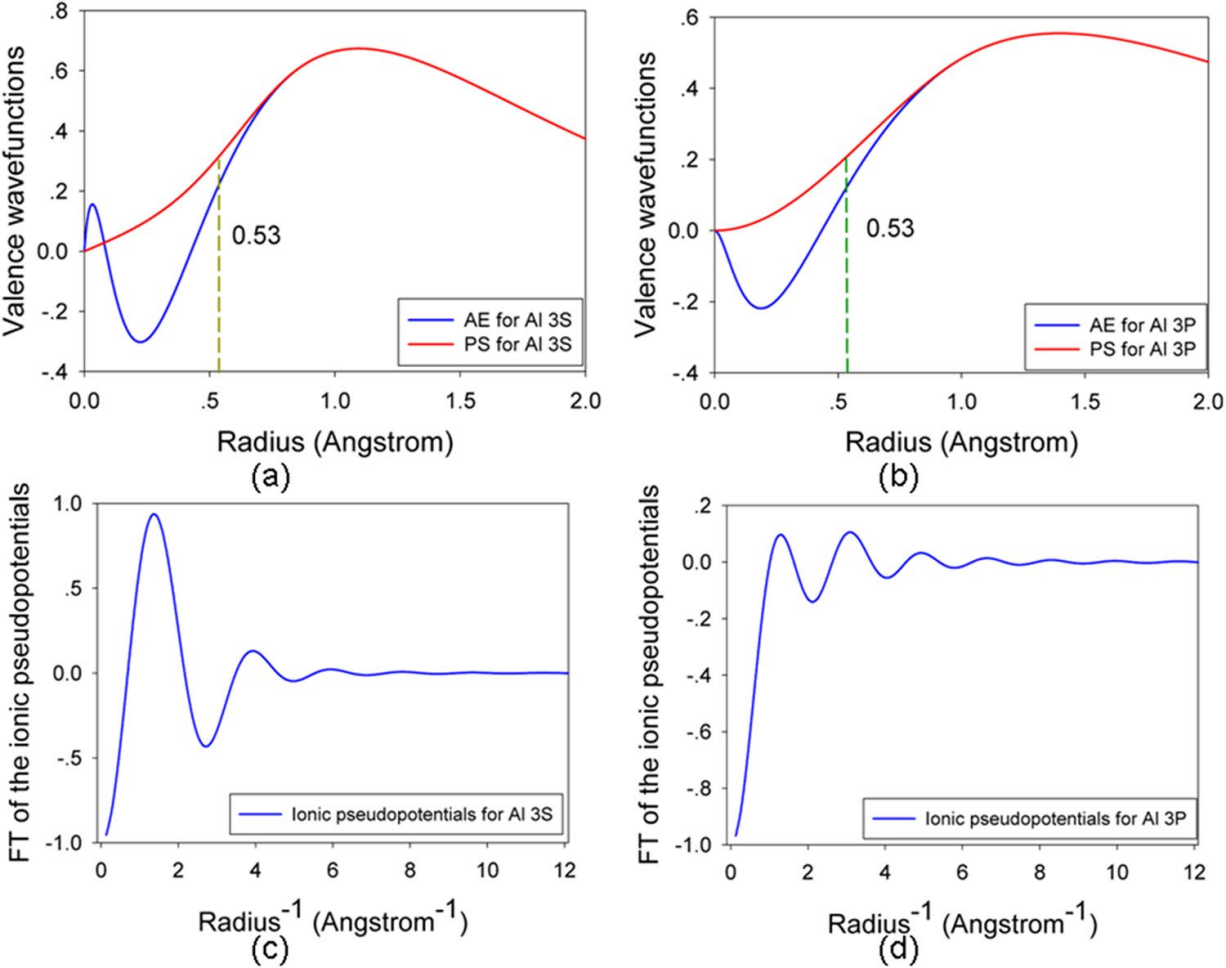
(**a**,**b**) All-electron (AE) and pseudo (PS) valence wave functions as a function of radius for aluminum 3 s and 3p orbitals; (**c**,**d**) Fourier transfer of the ionic pseudo potential as a function of radius^-1^ for aluminum 3 s and 3p orbitals.

**Figure 7:**
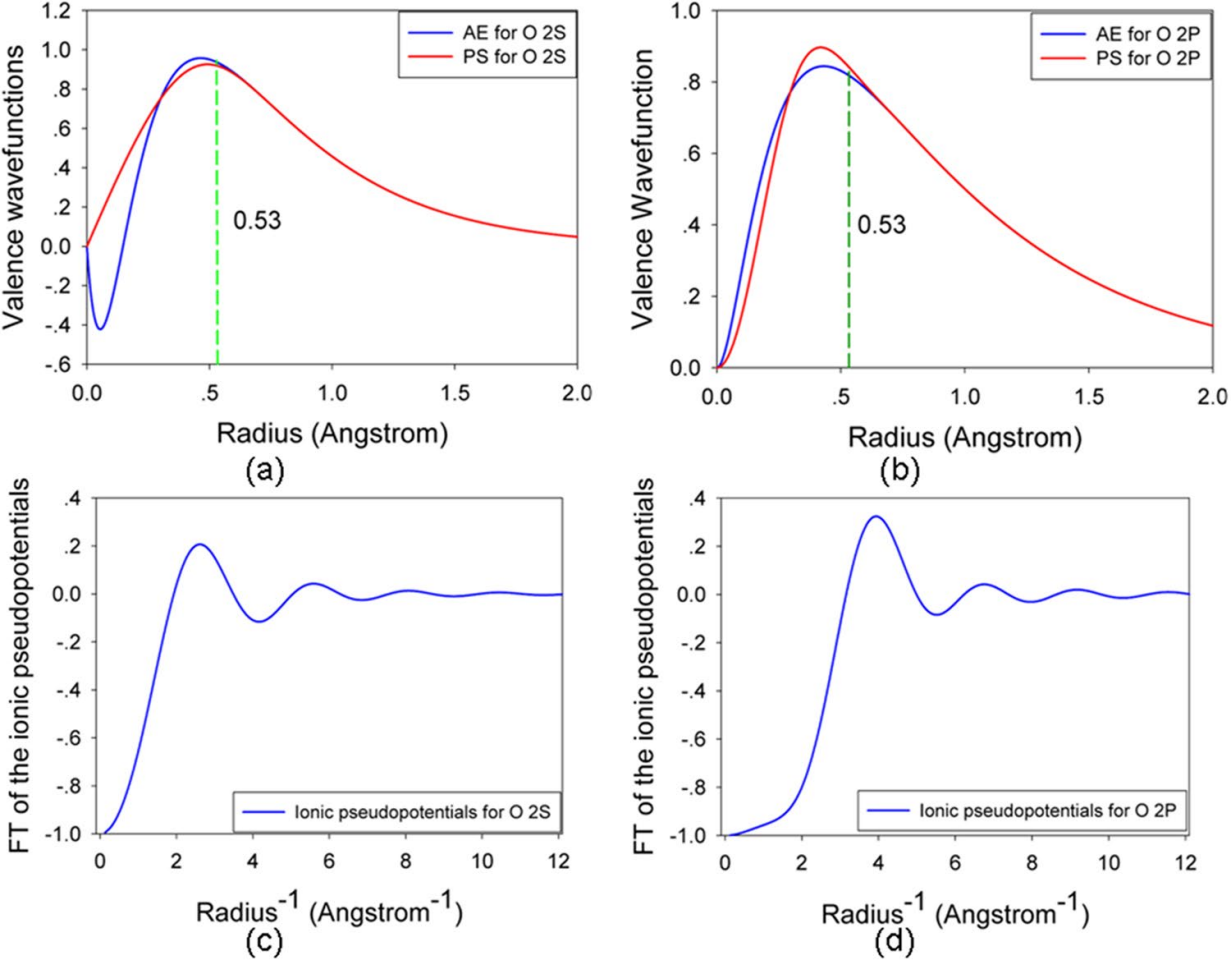
(**a**,**b**) All-electron (AE) and pseudo (PS) valence wave functions as a function of radius for oxygen 2 s and 2p orbitals; (**c**,**d**) Fourier transfer of the ionic pseudo potentials as a function of radius^−1^ for oxygen 2 s and 2p orbitals.

**Figure 8:**
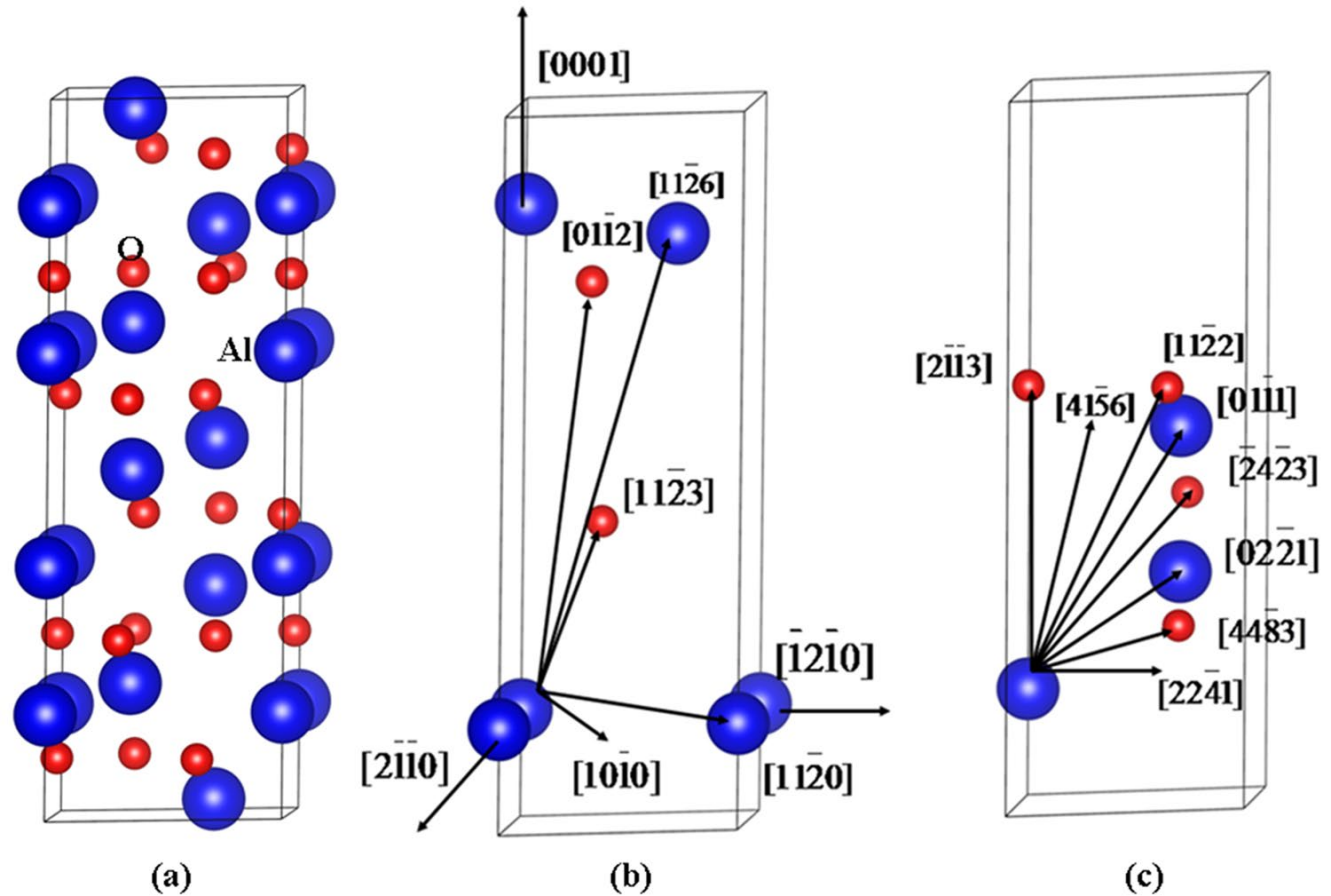
Illustration of schematic view of (**a**) α-Al_2_O_3_ structure; (**b**,**c**) incident directions in α-Al_2_O_3_.

All calculations are carried out using the Spanish Initiative for Electronic Simulation with Thousands of Atoms (SIESTA) code. The norm-conserving Troullier-Martins pseudopotentials^38^ are employed to determine the interaction between ions and electrons, and the exchange-correlation potential is described by the local-density approximation (LDA) in Ceperly-Alder parameterization^39^. The valence electron configurations are 2s^2^2p^4^ for O, with cutoff radii of 1.46 bohr for both orbitals. For Al the valence electron configurations are 3s^2^3p^1^, and the cutoff radii are 1.86 and 2.06 bohr for 3 s and 3p orbitals, respectively. The all electron and pseudo valence wave function and the Fourier transfer of ionic pseudo potential for Al and O are presented in Figs 6 and 7, respectively. As shown in Figs 6 and 7, when the cutoff radius is larger than 1.0 bohr, the pseudo valence wave functions for O and Al are in reasonable agreement with the all electron wave functions. A series of test calculations have shown that these relatively hard pseudopotentials can be used to describe the short-range interactions between atoms for recoil energy larger than 100 eV. The valence wave functions are expanded by a basis set of localized atomic orbits, and single-ζbasis sets (SZ) are employed, with a K-point sampling of 1 × 1 × 1 in Brillouin zone and a cutoff energy of 60 Ry. In the displacement events, sixteen directions for α-Al_2_O_3_, as illustrated in Fig. 8b and c, are taken into account. To simulate the low energy recoil events, a 3 × 3 × 1 supercell consisting of 270 atoms is used. The simulations are conducted with a NVE ensemble and a variable time step scheme is employed to avoid the instability of the system.
